# Undetected Barrett’s esophagus: how do we improve early detection?

**DOI:** 10.18632/oncotarget.28005

**Published:** 2021-11-23

**Authors:** Holli A. Loomans-Kropp, Ellen Richmond

**Keywords:** Barrett's esophagus, esophageal adenocarcinoma, screening, concurrent disease

Barrett’s esophagus (BE), a lesion that results from chronic gastroesophageal reflux disease (GERD), is the only known precursor of esophageal adenocarcinoma (EAC); however, BE lesions are rarely identified prior to EAC diagnosis [1, 2]. In our study investigating the association between usage of common pharmaceuticals and risk of EAC using Surveillance, Epidemiology, and End Results (SEER) Program Data linked to Medicare, we observed that a significant proportion of BE diagnoses occurred within one year of EAC (Figure 1), strongly suggesting prior missed EAC lesions [3]. Our findings concur with those established in the literature. For example, a recent retrospective cohort study found that 80% of EAC’s were diagnosed within one year of index endoscopy, while a meta-analysis showed that prevalence of concurrent BE and EAC was 56.6% (48.5–64.6%) [2, 4]. Recent studies have suggested that an increase in the number of endoscopic biopsies and use of advanced endoscopic modalities contribute to moderately better patient outcomes, though significant room for progress remains [2, 5, 6]. Clearly, better understanding the problem of concurrent diagnosis of BE and EAC is still needed to enable precise risk stratification of those likely to progress to EAC and identify apt candidates for cancer interception.

**Figure 1:**
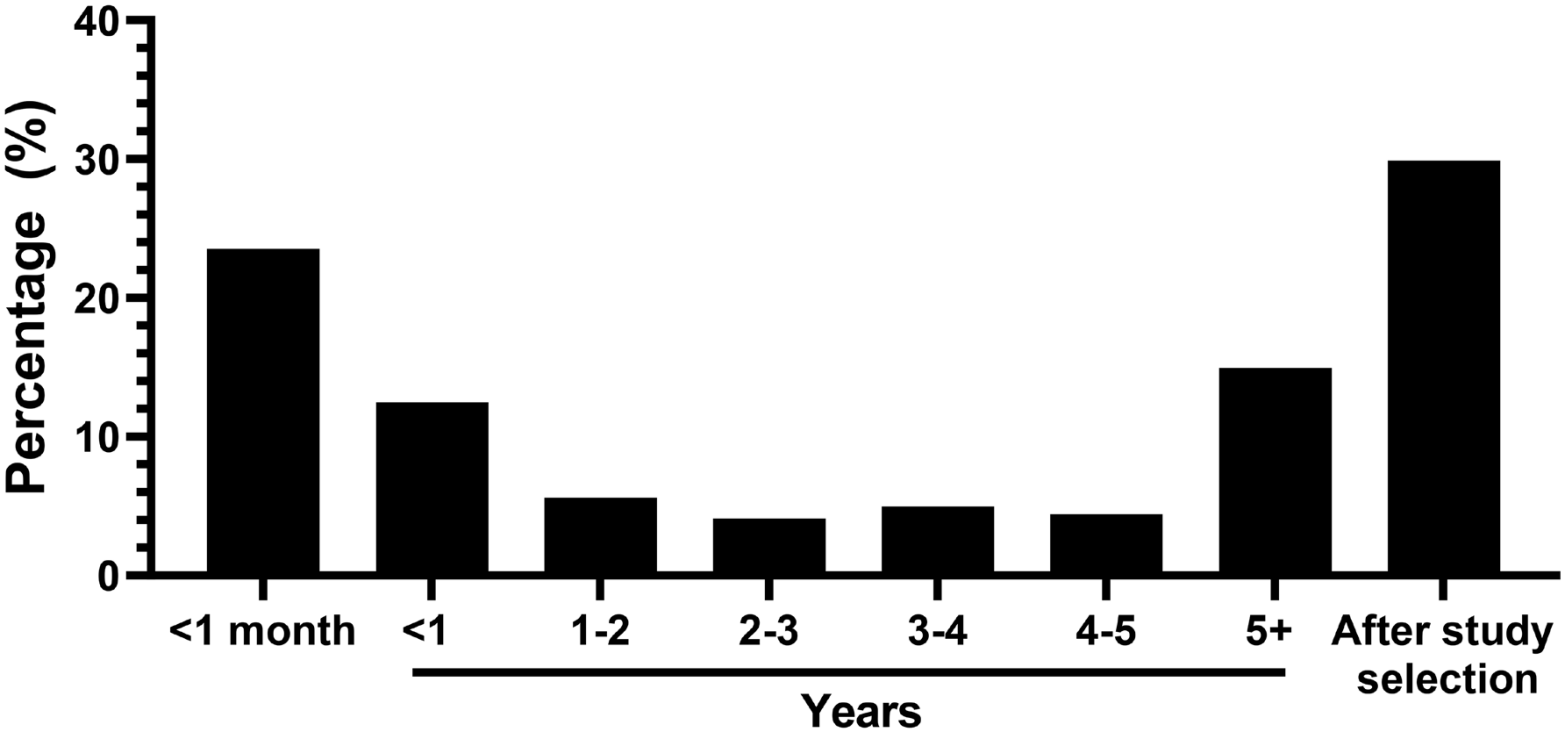
Time between BE and EAC diagnosis. Majority of BE diagnoses occurred within one month of EAC diagnosis or after the study selection in the SEER-Medicare data linkage (2007–2013).

Though several explanations for the observation of ‘concurrent disease’ have been proposed, we will discuss two dominant theories. First, it is suggested that individuals who first present for medical care with progressive mechanical dysphagia and weight loss (so-called ‘alarm symptoms’) and are diagnosed with late stage EAC with BE were likely asymptomatic or had atypical symptoms during initial disease progression. Such individuals, even in the presence of known risk factors (e.g., male sex, central obesity, older age, family history), are rarely referred for endoscopy because current societal guidelines for endoscopic screening for BE require chronic or frequent GERD symptoms [7]. In fact, unsuspected BE is not uncommon. A study conducting endoscopic esophageal evaluation in older males without known GERD found that approximately 25% of participants had detectable BE [8]. In compliance with current recommendations, these men would not have been diagnosed. Another recent study showed that the majority of BE cases without frequent GERD symptoms have at least one risk factor for BE and GERD, further suggesting the need for more flexible screening guidelines [6]. This illustrates a common dilemma to early cancer detection: screening at the population level is not cost-effective, but narrow recommendations do not capture those few at higher risk. To identify those who would benefit from endoscopic screening and/or treatment of pre-malignant lesions, (1) the expansion of BE screening eligibility criteria and (2) the development of less invasive approaches is necessary.

A second theory proposes that the widespread availability of effective over the counter (OTC) treatments for GERD may result in an underestimation of BE prevalence. Antacids and proton pump inhibitors, routinely used to alleviate GERD symptoms, can be acquired both through prescription or OTC [3, 9]. As such, acid suppression by proton pump inhibitors may relieve symptoms of GERD, possibly preventing individuals from seeking medical attention and thereby earlier detection of BE. This concern has recently been disputed in a study that showed that no significant difference between healthcare utilization of individuals with prevalent EAC (concurrent diagnosis of BE and EAC) compared all other EACs, as measured by the number of any type of medical office visits for patients with EAC (2). In our study, individuals with BE reported significantly more prescriptions for proton pump inhibitors than those without a diagnosis of BE, which could lower the number healthcare visits in the latter group [3]. However, data as to whether BE had been ruled out in the latter group (or data as to whether the latter group had undetected BE) was not available. Further complicating the equation is the fact that although BE results from esophageal exposure to gastric refluxate, BE is not an obligate outcome; only a small percentage of individuals with GERD develop BE. In a study of individuals with symptomatic GERD, only 5% of participants had endoscopically confirmed BE, compared to 2% of individuals without GERD [10]. Taken together, current evidence highlights the need for more precise approaches to screening to identify individuals at risk of BE or early-stage EAC [11, 12].

Improvements in the identification of people with BE will ultimately depend on advancements in BE screening practice which is currently expensive, invasive, and inconsistent [4, 13]. Upper endoscopy in individuals with GERD is the most common screening practice, though promising novel methods are being investigated. Tethered swallowable balloon capsules, Cytosponge™, and blood-based biomarkers, are in various stages of development [14–17]. Nevertheless, these new tools will need validation in large screening populations before they can become more economical for widespread use. This is a massive hill to climb, as the prevalence of BE risk factors are increasing and population-scale surveillance has, thus far, had limited value [18–20]. Improvements in the screening and surveillance process may emerge from (1) better risk stratification modeling to delineate low- and high-risk populations, (2) understanding the underlying genetic and epigenetic landscape of the disease, and (3) untangling gene-environment interactions.

As screening for BE becomes increasingly sensitive and specific, detecting clinically meaningful precursor lesions should become increasingly frequent. Further, the ongoing development of safer, minimally invasive screening modalities should lead to broader application. With this multidisciplinary approach, a decrease in the morbidity and mortality of EAC is on the horizon.

## References

[1] Hvid-Jensen F , et al. N Engl J Med. 2011; 365:1375–83. 10.1056/nejmoa1103042. 21995385

[2] Vajravelu RK , et al. Clin Gastroenterol Hepatol. 2021 Feb 5. 10.1016/j.cgh.2021.02.005. [Epub ahead of print]. 33549872 PMC8895727

[3] Loomans-Kropp HA , et al. Cancer Prev Res. 2021; 14:195–204. 10.1158/1940-6207.CAPR-20-0274. 32998939

[4] Tan MC , et al. Aliment Pharmacol Ther. 2020; 52:20–36. 10.1111/apt.15760. 32452599 PMC7293564

[5] Sawas T , et al. Clin Gastroenterol Hepatol. 2021 Apr 23. 10.1016/j.cgh.2021.04.032. [Epub ahead of print]. 33901662 PMC9799241

[6] Nguyen TH , et al. Clin Gastroenterol Hepatol. 2021 Apr 8. 10.1016/j.cgh.2021.04.008. [Epub ahead of print]. 33839273 PMC8900254

[7] Shaheen NJ , et al. Am J Gastroenterol. 2016; 111:30–50. 10.1038/ajg.2015.322. 26526079 PMC10245082

[8] Gerson LB , et al. Gastroenterology. 2002; 123:461–67. 10.1053/gast.2002.34748. 12145799

[9] Sheikh I , et al. Am J Gastroenterol. 2014; 109:789–94. 10.1038/ajg.2013.421. 24896751

[10] Fan X , et al. Dig Dis Sci. 2009; 54:572–77. 10.1007/s10620-008-0395-7. 18654849

[11] Shaheen NJ , et al. Ann Intern Med. 2012; 157:808–16. 10.7326/0003-4819-157-11-201212040-00008. 23208168

[12] Inadomi JM , et al. Ann Intern Med. 2003; 138:176–86. 10.7326/0003-4819-138-3-200302040-00009. 12558356

[13] Bhat SK , et al. Gut. 2015; 64:20–25. 10.1136/gutjnl-2013-305506. 24700439

[14] Tan WK , et al. Gastrointest Endosc Clin N Am. 2021; 31:43–58. 10.1016/j.giec.2020.08.004. 33213799

[15] Spechler SJ , et al. Gastroenterology. 2018; 154:1594–601. 10.1053/j.gastro.2018.03.031. 29577931

[16] Wenker TN , et al. Dig Dis Sci. 2018; 63:3112–19. 10.1007/s10620-018-5241-y. 30109579 PMC6185782

[17] Hur C , et al. Cancer. 2013; 119:1149–58. 10.1002/cncr.27834. 23303625 PMC3744155

[18] Yamasaki T , et al. J Neurogastroenterol Motil. 2018; 24:559–69. 10.5056/jnm18140. 30347935 PMC6175565

[19] Bohmer AC , et al. Neurogastroenterol Motil. 2017; 29:e13017. 10.1111/nmo.13017. 28132438

[20] Macdonald CE , et al. BMJ. 2000; 321:1252–55. 10.1136/bmj.321.7271.1252. 11082084 PMC27527

